# Health information system strengthening and malaria elimination in Papua New Guinea

**DOI:** 10.1186/s12936-017-1910-0

**Published:** 2017-07-05

**Authors:** Alexander Rosewell, Leo Makita, David Muscatello, Lucy Ninmongo John, Sibauk Bieb, Ross Hutton, Sundar Ramamurthy, Phil Shearman

**Affiliations:** 1 PNG Remote Sensing Centre, PO Box 1733, Waterfront, Konedobu, Port Moresby, Papua New Guinea; 2 School of Public Health and Community Medicine, University of New South Wales, Sydney, 2052 Australia; 3 National Department of Health, Port Moresby, Papua New Guinea; 4 Port Moresby, Papua New Guinea; 5 School of Botany and Zoology, The Australian National University, Linnaeus Way, Canberra, 0200 Australia

## Abstract

**Background:**

The objective of the study was to describe an m-health initiative to strengthen malaria surveillance in a 184-health facility, multi-province, project aimed at strengthening the National Health Information System (NHIS) in a country with fragmented malaria surveillance, striving towards enhanced control, pre-elimination.

**Methods:**

A remote-loading mobile application and secure online platform for health professionals was created to interface with the new system (eNHIS). A case-based malaria testing register was developed and integrated geo-coded households, villages and health facilities. A malaria programme management dashboard was created, with village-level malaria mapping tools, and statistical algorithms to identify malaria outbreaks.

**Results:**

Since its inception in 2015, 160,750 malaria testing records, including village of residence, have been reported to the eNHIS. These case-based, geo-coded malaria data are 100% complete, with a median data entry delay of 9 days from the date of testing. The system maps malaria to the village level in near real-time as well as the availability of treatment and diagnostics to health facility level. Data aggregation, analysis, outbreak detection, and reporting are automated.

**Conclusions:**

The study demonstrates that using mobile technologies and GIS in the capture and reporting of NHIS data in Papua New Guinea provides timely, high quality, geo-coded, case-based malaria data required for malaria elimination. The health systems strengthening approach of integrating malaria information management into the eNHIS optimizes sustainability and provides enormous flexibility to cater for future malaria programme needs.

## Background

Malaria remains a killer globally, accounting for an estimated 14 million cases and 438,000 deaths [1]. Papua New Guinea has among the highest malaria transmission rates in the Western Pacific, accounting for 71% of cases and 77% of deaths [1]. Malaria epidemiology is heterogeneous below 1400 m, with a risk of seasonal outbreaks identified between 1400 and 1650 m [2]. Despite this high burden, the malaria control programme has reported important successes in recent years, including a 75% decline in malaria admissions since 2000, and a decline in parasite prevalence from 12.4 to 1.8% between 2009 and 2014 [1]. While these declines have been linked to the increased coverage of long-lasting insecticidal nets (LLIN) and the increased proportion of confirmed cases treated with artemisinin-based combination therapy (ACT) [1], interpretation must consider the introduction and national roll-out of rapid diagnostic tests (RDTs) from 2011. Previously, febrile patients were reported as malaria through the National Health Information System (NHIS), with only ~15% tested for malaria infection by RDT and 3.6% tested by microscopy [3].

In 2015, the World Health Organization (WHO), released the Global Technical Strategy for Malaria 2016–2030, emphasizing the critical need to transform malaria surveillance as a core intervention [4]. At this time, Papua New Guinea became the 17th member of the Asia Pacific Malaria Elimination Network, and endorsed the Asia Pacific Leaders Malaria Alliance, Malaria Elimination Roadmap by 2030. Papua New Guinea will strive towards malaria elimination in selected settings, once considered feasible following the adoption of new tools, in combination with health systems strengthening [5].

Strong surveillance systems linked to effective responses are critical for malaria elimination [6]. In elimination settings, data collection, analysis, reporting, active case finding, and linkage to response must happen quickly to identify infections (symptomatic and asymptomatic), prevent ongoing transmission, and decrease transmission efficiency of vectors [6]. For elimination, case reporting needs to transition from periodic and aggregated at sub-national levels to reporting of individual geo-located cases in real-time [6]. Systems must enable complete and rapid case notification, integration of related datasets such as population data, central data storage and management with widespread access to decision makers, automated analyses and customized reports and workflows that lead to timely and targeted responses [6]. Systems should also track and map cases over time using spatial information. Continued transmission monitoring, outbreak detection and the identification of residual hotspots have all been highlighted as requirements for malaria elimination in Papua New Guinea [2]. Globally, there are few malaria information systems meeting the real-time information needs of malaria control programmes [6], including in Papua New Guinea [7].

Investment in information technologies that drive real-time surveillance systems and effective response are essential for malaria elimination [6]. m-health has recently demonstrated its potential for strengthening disease surveillance in Papua New Guinea [8, 9]. The Strengthening Rural Primary Health Services Project [10] includes a pilot of mobile technologies and geographic information system (GIS) in the capture and reporting of NHIS data (eNHIS).

## Aim

To describe an initiative to strengthen malaria surveillance in a 184-health facility, multi-province, project aimed at strengthening the National Health Information System in a country with fragmented malaria surveillance, striving towards enhanced control.

## Methods

### Existing systems for malaria information management

#### Malaria programme system

Health services in Papua New Guinea are the primary responsibility of the State through a decentralized public management system. The National Malaria Control Programme strategy is set by the National Department of Health, Division of Public Health, Section of Malaria and Vector-Borne Disease. The current National Malaria Strategic Plan 2014–2018 focusses on control rather than elimination. The National Programme contributes advisors for the four regions of the country and is responsible for the procurement and distribution of bed nets, malaria drugs and diagnostic supplies.

The provincial governments are responsible for programme implementation through their Divisions of Health or the Provincial Health Authority. While many provinces have a Disease Control Officer or Malaria Supervisor, intervention mostly occurs in the primary health care setting by nurses and community health workers at rural aid posts and health centres. This involves passive case detection as symptomatic patients present to health facilities and are diagnosed with RDTs and treated. A July 2016 mid-term review of the programme has recommended that a push to elimination should be commenced, particularly in island provinces and communities where feasible.

#### NHIS monthly summary report

Paper-based monthly summary reports are submitted by health facilities to the Provincial Health Office. Summary reports are entered into a Microsoft Visual FoxPro 7.0 database (Microsoft Corporation, Seattle, WA, USA). Hard-copy and electronic data are sent to national level, where they are re-entered and cleaned before a national dataset is finalized. The paper-based monthly health centre summary report contains the aggregated number of: (1) clinical and confirmed (RDT or microscopy) outpatient malaria cases and inpatient cases and deaths, by age group (0–4, 5–14, 15+ years), pregnancy status and gender; (2) malaria cases by diagnostic procedure, gender, age group (as above) and *Plasmodium* species, including total tested; and, (3) ACT courses by outpatient/inpatient status. Stock-out information includes chloroquine and amodiaquine (both now obsolete), Fansidar^®^, ACT, quinine injection, primaquine, and RDT. Malaria data collected by health workers or village volunteers during active case finding at village level may be integrated into the paper-based system through aid-post reporting. Monthly outpatient count data are aggregated from daily tally sheets by facility health staff. Monthly inpatient count data, including fatal outcomes, are aggregated from the inpatient register, discharge register and malaria testing register. Monthly summary reports are taken to the Provincial Health Office and entered into the National Health Information System’s FoxPro database (Microsoft Corp, Redmond, WA, USA), before electronic and hard copies are sent to national level for re-entry, cleaning and usage. Monthly summary reporting is commonly delayed by ~3 months, sometimes much longer [9]. Microsoft FoxPro software is no longer supported [11]. Inpatient data from rural health facilities is coded by two staff at national level using a country-specific shortlist of ~450 International Classification of Diseases (ICD) codes. Malaria specific codes include: B50-*Plasmodium falciparum* malaria, B500-Cerebral malaria, B51-*Plasmodium vivax* malaria, B52-*Plasmodium malariae* malaria, B54-Malaria (severe unspecified). Inpatient discharge data coding is currently delayed by ~2–3 years due to entry backlog [9]. All data are accessible for analysis by one person at provincial level, the Provincial Health Information Officer, and Information Officers within the monitoring, evaluation and research branch of the national authorities, but not directly by the national malaria programme. Stakeholder feedback is in the form of the hard copy, NHIS annual report, which is sent to district and provincial health offices by ~May of the preceding year.

#### Malaria testing registers

All febrile patients presenting at a health facility are tested for malaria and recorded in a paper-based register. Data capture includes: date, gender, age, result, treatment name. In highland areas, clinician discretion is used before testing, based on travel history and other factors. Malaria testing register copies are sent to the Provincial Health Office; however, there is no computer-based system for data entry and analysis. Anecdotally, these data are not routinely analysed.

#### Outbreak detection

Outbreaks are reported through the event-based surveillance system [12]. Currently there is no policy, procedure or information system to detect outbreaks using routine data.

#### Population

Health facility catchment, district and provincial population data are disseminated to the provincial health offices from the national health authorities based on an extrapolation from the previous census, with the option for local modification. Population data accuracy is a concern [13].

### New systems for malaria information management (eNHIS)

#### Mobile application and database

A mobile application was created for healthcare workers to interface with the new system. The password-protected application includes modules for data entry, automatically generated summary data, national and international guidelines (including the WHO), a data dictionary, and an automatically updated contact list. The application was developed as an android package kit (APK) that runs on the android platform, with data transmission to the server and synching between tablets and the server occurring via 2G and 3G mobile telephone networks of both national mobile telecommunications providers. Application version updates are performed either directly or remotely. Administrators can remotely clear all data on a tablet at any time (i.e., if stolen). Future amendments to the system will include duplicate management algorithms, and could include the mapping of both notification data and bed net distribution to the household level and the capture of G6PD test results. Data storage on the in-country server is in line with national standards for health information management, including confidentiality and redundancy.

#### NHIS monthly summary report

Monthly outpatient count data are aggregated automatically from the daily tally sheets in the mobile reporting application. Malaria data collected during active case finding at village level has the potential for integration into the system through future aid-post reporting in the mobile reporting application or alternative approaches that enable seamless data integration. Monthly inpatient count data, including fatal outcomes, are aggregated automatically from the inpatient discharge register in the mobile reporting application. All data are accessible for analysis at all levels of the health system, from the moment the data are entered into a tablet with server-connectivity. An ICD-coding tool for inpatient visits was integrated into the mobile reporting application to enable remote health facility staff to code inpatient discharge data using the national shortlist of ~450 ICD codes. Additional variables include: village (selected from a geo-coded list of ~20,000 villages, or entered free text if not listed) and date of discharge.

#### Online platform

**Fig. 1:**
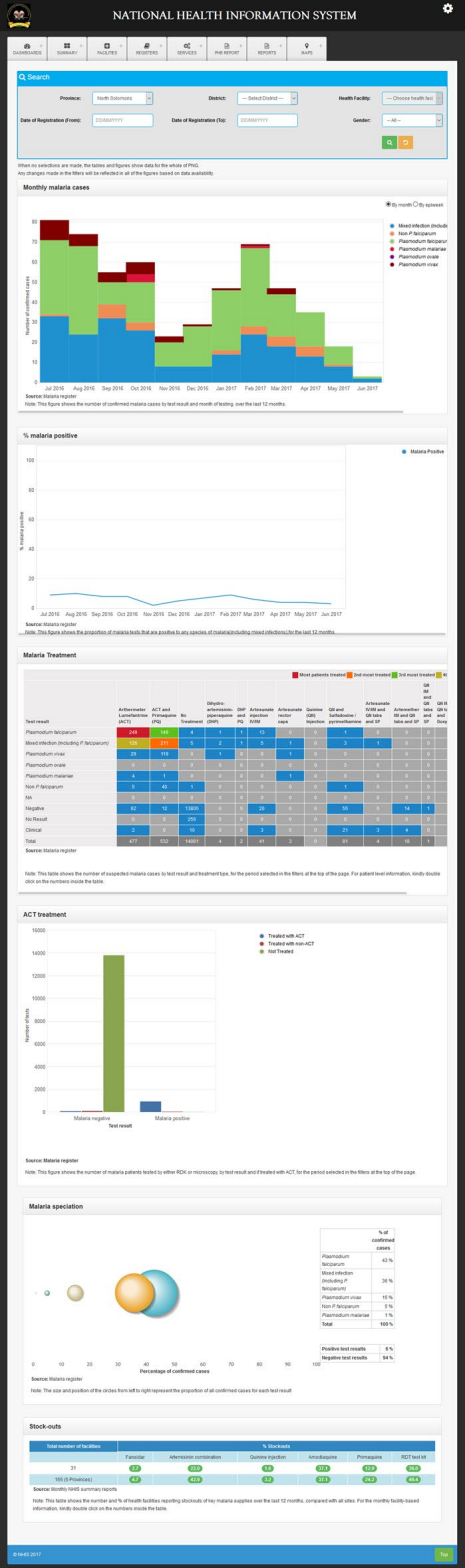
Malaria program management dashboard (selected drill down tools)

**Fig. 2:**
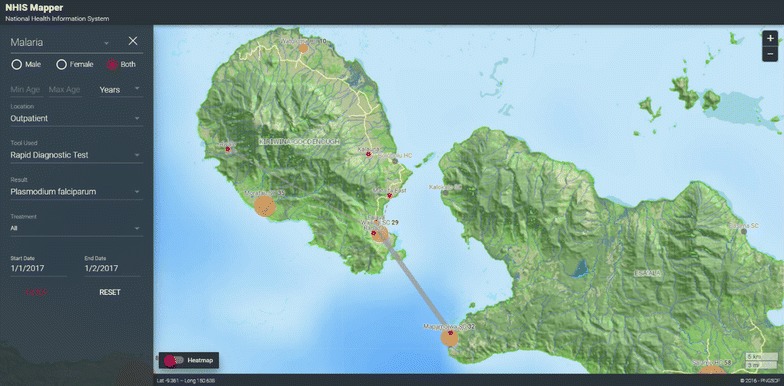
Map of village of residence of confirmed malaria cases, per health facility

**Fig. 3:**
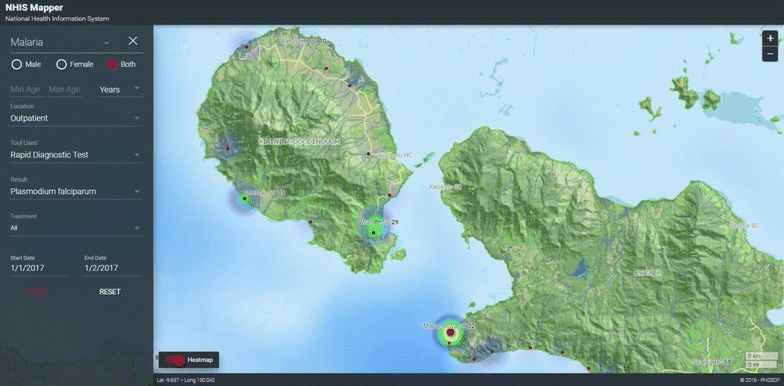
Heatmap of malaria transmission foci

**Fig. 4:**
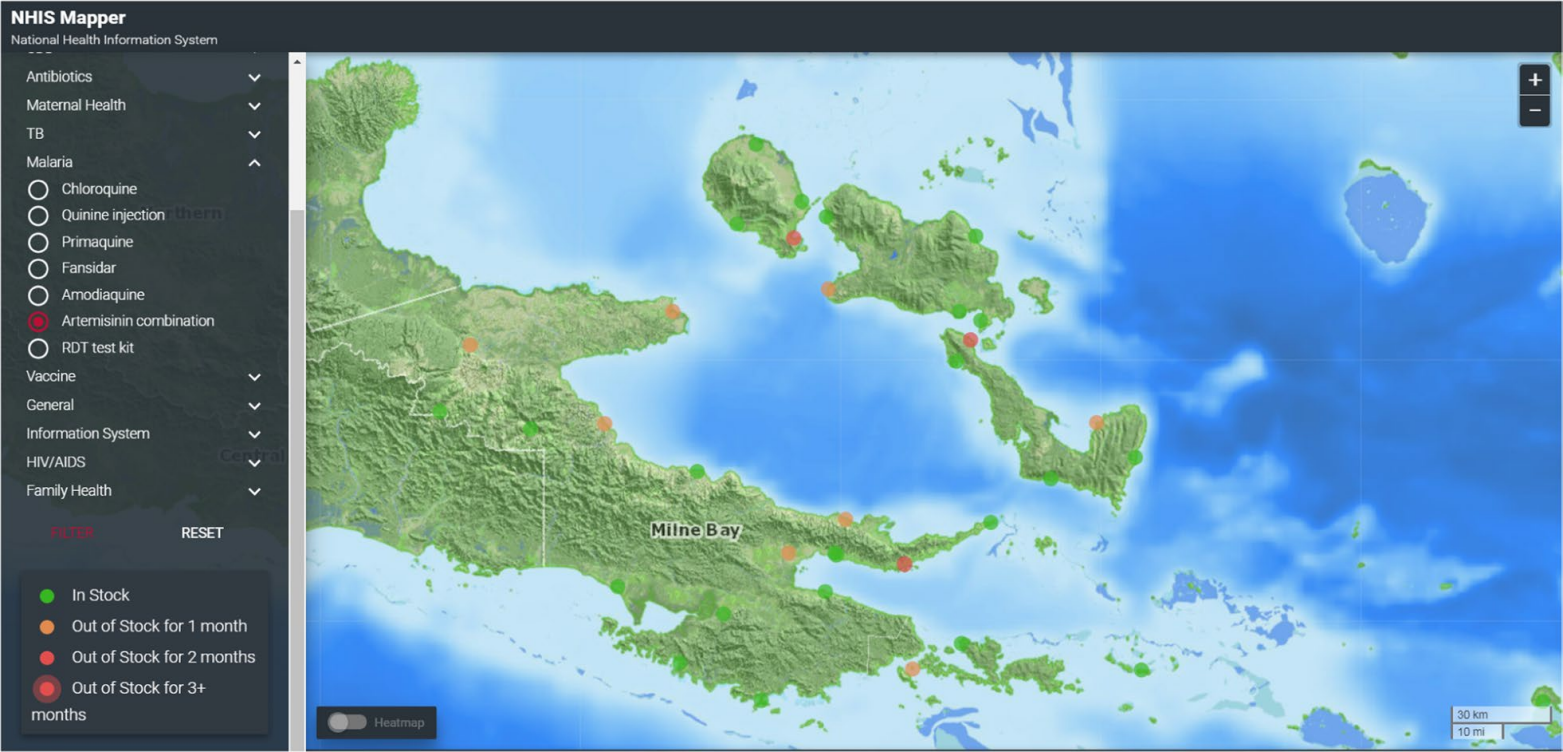
Shortages of artemisinin combination therapy

A javascript platform was used as the interface, standardized query language (SQL) server for a back-end database, VMware as the back-end operating engine, and a proprietary GIS platform for the mapping interface, in line with information communication technology standards of the national health authorities [9]. A malaria testing register was integrated into the eNHIS with additional variables including: first name, last name, village, and clinic setting. Programme management dashboards were created for malaria (Fig. 1) and GIS-mapping of all data and indicators (Figs. 2, 3, 4).

#### Outbreak detection

In addition to outbreaks identified through event-based surveillance, statistical algorithms that aimed to identify malaria outbreaks at village, health facility, district, and provincial level were implemented. These algorithms considered the local epidemiology of malaria where malaria transmission is highly endemic at low altitudes, but with a risk of outbreaks at higher altitudes. A major challenge in this low-resource setting is timeliness, completeness and accuracy of reporting. Simple algorithms that did not depend on a long baseline of complete reporting and that did not assume complete reporting were chosen. Reporting facilities were classified as endemic or non-endemic. For non-endemic reporting sites and regions, a simple algorithm is currently under development. For endemic sites, a method was required that compared an observed indicator of malaria with an expected value that was less sensitive to changes in reporting completeness. A method based on the observed proportion of malaria tests that were positive and compared this with the expected proportion positive was chosen. The expected proportion used a short baseline of four weeks to limit the impact of changing completeness over time. The short baseline had the additional benefit that the expectation would include current within-season data, and compensate, to some extent, for slow moving seasonal changes in incidence. For the malaria testing register, the 95% confidence interval of the proportion of laboratory specimens that are positive for malaria in the most recent week was compared with the specific proportion positive in the previous four weeks. If the lower confidence limit of the most recent week is above the proportion in the prior four weeks then a signal occurs.

To ensure robustness in a wide range of proportions, exact methods are used for the calculation of confidence intervals [14]. The equations for the algorithm are:

$$ Proportion = n_{Positive} /n_{Tested} $$

where $n_{Positive}$ is the number of specimens with a collection date in the 7 days prior to the report generation date, and $n_{Tested}$ is the total number of specimens collected in the same period.

A signal occurs if the lower 95% confidence limit of *Proportion* exceeds

$$ N_{Positive} /N_{Tested} $$

where $N_{Positive}$ and $N_{Tested}$ represent the count of positive and tested specimens collected in the 28 days prior to the most recent 7 days. The lower confidence limit of *Proportion* is calculated as [14]:

$$ \frac{{n_{Positive} }}{{n_{Positive} + \left( {n_{Tested} - n_{Positive} + 1} \right)F_{{0.025,   2n_{Tested} - 2n_{Positive} + 2,   2n_{Positive} }} }} $$

where $F_{{0.025,   2n_{Tested} - 2n_{Positive} + 2,   2n_{Positive} }}$ represents a value from the F distribution with a right-tailed probability of 0.025, a numerator degrees of freedom of $2n_{Tested} - 2n_{Positive} + 2$ and a denominator degrees of freedom of $2n_{Positive}$.

**Fig. 5:**
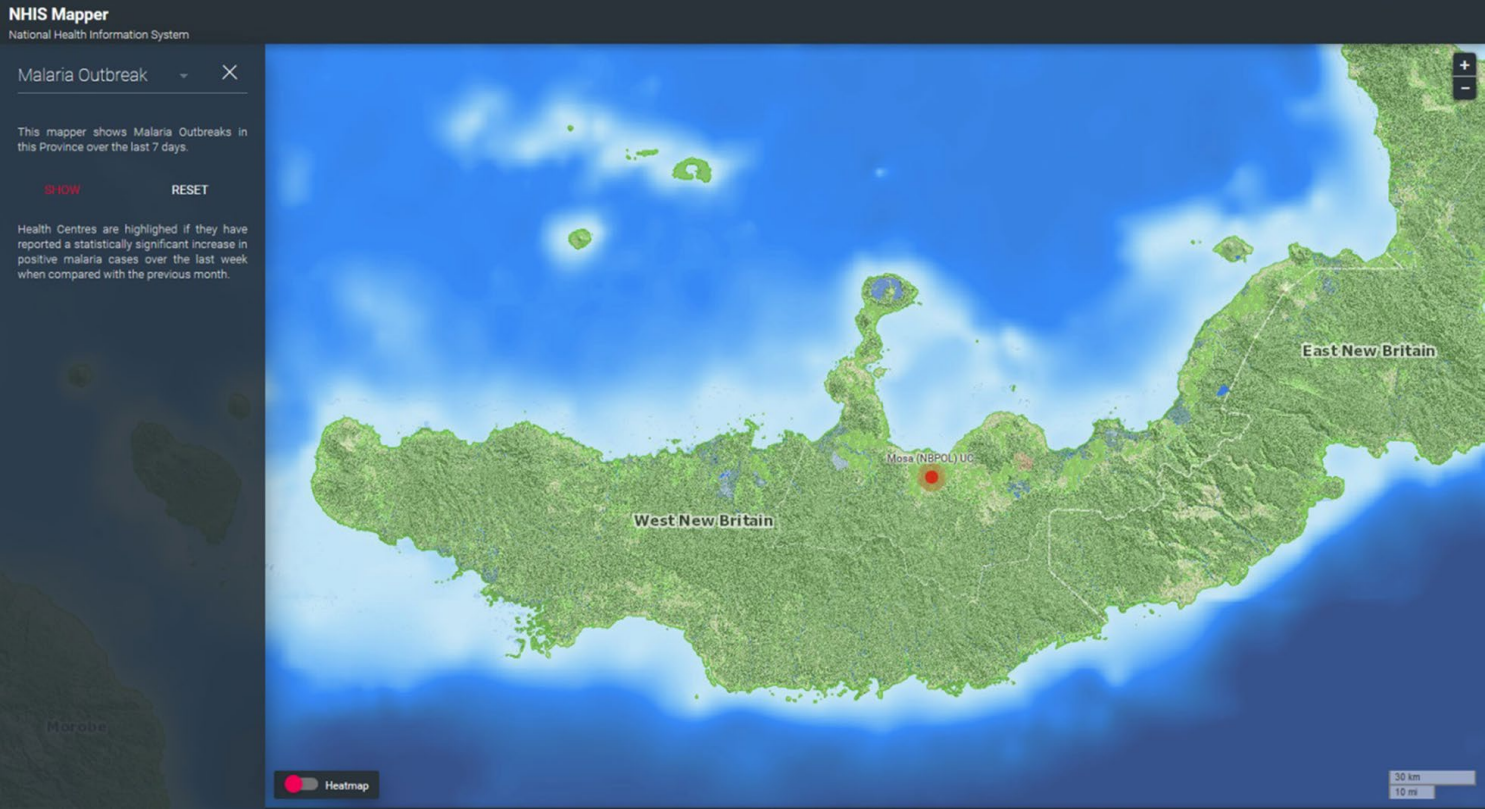
Algorithm based malaria outbreak detection mapping tool

Statistical algorithms for outbreak detection down to village level were integrated into the mapping platform (Fig. 5).

### Data analysis

Descriptive analyses of malaria register data were performed using STATA/IC 14 (Stata Corp., College Station, TX, USA). Ranges and inter-quartile ranges were calculated using the *summarise*, *detail* function.

## Results

Since its inception in 2015, 160,750 malaria testing records, including village of residence, have been reported to the eNHIS. These case-based, geo-coded malaria data are 100% complete, with a median data entry delay of 9 days from the date of testing (interquartile range 3–21 days; range 0–626 days). The system maps malaria to the village level as well as the availability of treatment and diagnostics to health facility level. Data aggregation, analysis, outbreak detection, and reporting are automated.

## Discussion

This paper describes recent enhancements to malaria surveillance in a country slowly moving towards malaria elimination, within the context of strengthening the National Health Information System in a country with fragmented malaria surveillance. With the eNHIS, malaria case reporting has shifted from aggregated sub-national reporting to individual geo-located cases reporting and is timely and complete. All malaria control stakeholders can access the data and simple to use programme management tools. All data can be mapped to health facility or village level so that transmission foci can be visualized and responses targeted. Data aggregation, analysis, outbreak detection, and regular reporting are automated.

Malaria outbreak detection systems using statistical algorithms have been implemented across a range of settings [15–18]. The eNHIS has made available geo-coded malaria notifications that can now be used for timely outbreak detection for the first time. Because alert detection operates prospectively using the latest data, it is important to design and evaluate alert algorithms in a real-world context to accommodate actual reporting delays, accuracy limitations and incompleteness. This is a major challenge in this setting and limits the choice of algorithms to ones that adapt to data available rather than those that assume that reporting completeness is high and constant. The proportion positive is not dependent on complete reporting. The short baseline comparison period reduces dependency on complete reporting over long periods. Additionally, for simplicity, statistical tests were not included in the algorithm such as Chi square tests for comparison of proportions. This is because the proportion based on the number of tests included in the most recent observation week would typically be based on a much smaller number of tests and would be more unstable than the proportion in the previous four weeks, which is used for the expected proportion in the observation week. A somewhat similar approach, called the ‘slide positivity percentage’, was applied retrospectively, along with other algorithms, by Teklehaimanot et al. using Ethiopian malaria surveillance information. The slide positive percentage performed no worse than the other algorithms in that study. Other algorithms suitable in this setting were not considered because they were based on case counts rather than proportions, and would not be suitable for incomplete data. Another disadvantage of the methods assessed by Teklehaimanot et al. is that they used expectations based on data collected for up to 10 years and thus would require completeness over long periods. They also included future years in the estimation of the threshold for a given year, which is not realistic in an applied prospective application. They did argue, however, for deliberately simple approaches considering the limited capacity in low-resource contexts [18]. In addition to detecting outbreaks, the eNHIS can provide timely information on supply needs (treatment and test kits), help monitor transmission and mortality, and support the evaluation of interventions during outbreaks.

Current malaria elimination interventions aim to reduce the asymptomatic reservoir through mass screening and treatment and mass drug administration with ACT [19]. As the next generation tests for screening asymptomatic reservoirs and novel treatment regimens are adopted (e.g., single dose tafenoquine) [20], and glucose-6-phosphate dehydrogenase (G6PD) monitoring is implemented, relevant data captures can now be immediately updated in the mobile reporting application rather than waiting for changes to paper-based data captures. Full adherence to malaria treatment protocol by healthcare workers has previously been identified as problematic in Papua New Guinea [3]. With the eNHIS, the malaria programme management dashboard now enables timely monitoring of treatment protocol adherence (Fig. 1). Access to first-line anti-malarial treatment can be problematic [19], so in future, automated detection of health facility level stock-outs of malaria items (treatment and RDTs) may enable a more timely response. Strengthened health information system data have also demonstrated their utility for evaluating the scale-up of malaria interventions [21], which should now be possible with the eNHIS.

Malaria risk mapping is an essential component of efficient resource allocation in elimination settings [22]. Spatial technologies are providing unprecedented opportunities for rapid risk assessment in malaria-endemic areas [23] by overlaying ecological data relevant to mosquito breeding and transmission, notification data and access to health services. These technologies enable the development of decision support systems for more accurate and timely responses [24], including active case detection through cluster surveys and timely case follow-up. The geo-coded databases of ~20,000 villages, all households and health facilities embedded in the eNHIS as well as the timely clinical and laboratory data, provide enormous potential to automate risk assessment and response in the near future.

**Fig. 6:**
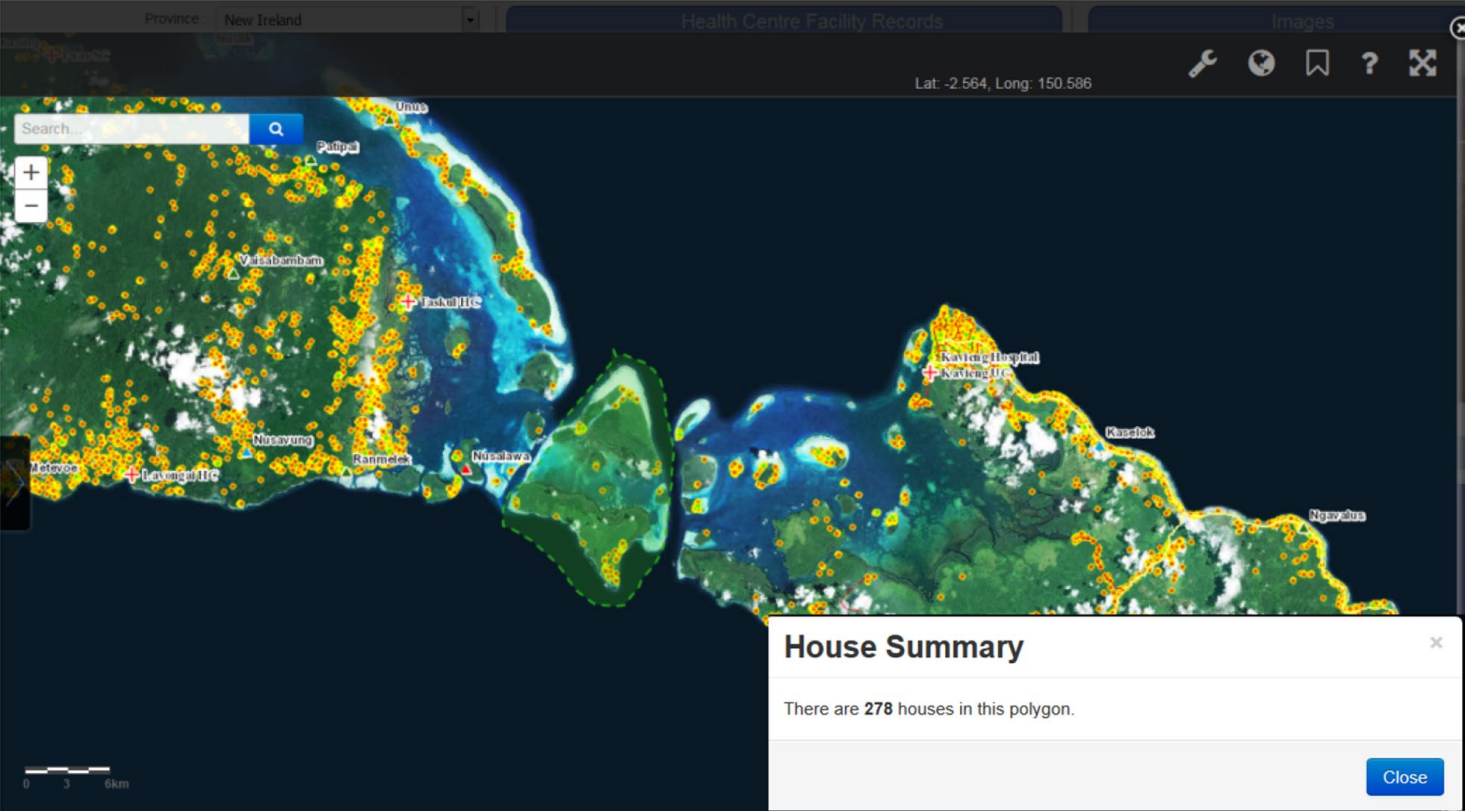
Bed net distribution tool, using geo-coded households

In Papua New Guinea, vector control, including LLINs, has had a significant impact on malaria transmission, species composition and feeding behaviour [25]. LLIN distribution campaigns are operationally challenging, with coverage gaps recently identified. Monitoring progress in bed net coverage has been hampered by the lack of reliable up-to-date population figures of census units [26], so that village level population censuses are required prior to distribution [26]. National household mapping within the eNHIS will soon be used to strengthen bed net distribution campaign microplanning. In future, village-level bed net data could be integrated to enable coverage mapping and analysis alongside other control and elimination indicators, as well as alternative population estimates. The impact of increased LLIN and treatment of pregnant women is likely decreasing rates of maternal anaemia and low birth weight babies [27], however timely quantification of the impact is required [28], all of which should now be achievable with the eNHIS. Bed net distribution microplanning may soon benefit from a tool that integrates household composition data with GIS data from mapping every household in the country using high resolution satellite imagery (Fig. 6). The availability of geo-coded, village-level, malaria data may enable the use of routine malaria data to determine health facility catchment populations, which has major service delivery implications [29].

WHO guidelines for malaria elimination strongly recommend malaria surveillance through strengthening their disease surveillance, health information and vital registration systems [30]. However, malaria programmes frequently receive significant donor support to build malaria surveillance systems that are not aligned with the national health information systems [31, 32]. In fact, a recent paper on malaria surveillance systems to facilitate elimination makes no mention of strengthening health information systems as a way to achieve malaria elimination [33]. Evidence suggests there is a dramatic skewing of Global Fund investments in health system-strengthening away from health information system-related interventions [34]. These findings from Papua New Guinea should provide sufficient field evidence that health system strengthening can support countries to achieve malaria elimination goals within fragmented systems, while also enabling enormous opportunities to improve health outcomes more broadly. However, effective health system strengthening relies on collaboration between agencies and national commitment as well as concerted partnership with all health system stakeholders [34], something that is yet to be achieved in Papua New Guinea.

## Conclusions

This work demonstrates that using mobile technologies and GIS in the capture and reporting of NHIS data in Papua New Guinea provides timely, high quality, geo-coded, case-based malaria data required for malaria elimination. The health systems strengthening approach of integrating malaria information management into the eNHIS optimizes sustainability and provides enormous flexibility to cater for future malaria programme needs.

## Abbreviations

LLIN: long-lasting insecticidal nets
RDT: rapid diagnostic tests
NHIS: National Health Information System
WHO: World Health Organization
ICD: International Classification of Diseases
APK: android package kit
SQL: standardized query language
G6PD: glucose-6-phosphate dehydrogenase
GIS: geographic information system
